# The sustained usefulness of online learning to educate nurses about antibiotic stewardship

**DOI:** 10.1017/ash.2024.3

**Published:** 2024-01-30

**Authors:** Mary Catanzaro, Lauren Geary

**Affiliations:** Quality Initiatives Department, The Hospital and Healthsystem Association of Pennsylvania, Harrisburg, PA, USA

## Background

In 2020, a series of three online learning modules to improve nurses’ knowledge of antimicrobial stewardship (AS) were developed and launched at a large academic medical center to integrate nursing into the antibiotic stewardship program. Literature review at that time suggested that in addition to hospital culture, lack of knowledge about antibiotics and the nurse’s role in stewardship could be reasons for poor engagement in stewardship activities including bedside rounding.^1,2^

Antimicrobial stewardship involvement was an expectation set by hospital leadership and was in place for several years. The decision to bolster education of nursing staff was an intentional move to advance stewardship at the bedside. In-person education was offered, but challenges in staff schedules proved to be a barrier. The use of online learning allowed nurses to learn at their own pace and gave them the flexibility to access modules at any time and location. It was mandatory for all nurses and after initial launch, became part of orientation for new staff. As part of the orientation process, modules helped to set baseline knowledge and expectations with no additional workload to the pharmacy staff, which had spearheaded previous educational efforts.

We compared results of the surveys taken on initial launch of the modules^3^ to those taken by new hires over the next two years and did not expect there to be any significant differences.

## Method

To address the nurses’ knowledge gap, modules were built with an e-learning platform and focused on general stewardship principles, appropriate specimen collection, interpretation of laboratory results, review of the antibiogram, understanding the difference between colonization and infection, interpretation of gram stains, minimal inhibitory concentrations for antibiotics, the Centers for Disease Control and Prevention Core Elements of a Successful Stewardship Program, how antimicrobial resistance develops, the difference between allergic reaction and side effects, how to take an allergy history, intravenous to oral medication conversion (IV to po), rationale behind de-escalation of antibiotics, and pharmacokinetics–pharmacodynamics. Modules were built with the intent to encourage and empower participation in patient rounds or discussions with physicians. The modules also provided a fundamental understanding of topics that could prove useful regardless of experience, unit work, or sustained interest in stewardship activities. The entire series took 75 minutes to complete.

An online Survey Monkey link immediately followed the final module and was used to assess the usefulness of the modules as well as the nurses’ attitudes toward participation in stewardship activities. Information was collected regarding how long the respondent has practiced nursing, participation in unit rounds, and perceived barriers to participation in stewardship activities.

We continued to collect data (through the Survey Monkey link) for nurses who viewed the modules over the next two years for later analysis. After the initial launch, only newly hired nurses (*n* = 213) were required to take the course.

## Results

Comparison of data (initial-launch nurses to post-launch nurses, see Table 1) showed no significant differences across barriers, overall usefulness, or nurses with units using multidisciplinary rounds. During the pandemic, the years of experience in nursing (4–6 and >10 years) changed significantly (*P* = .03 and .024, respectively), as well as nurses comfort level for participation in multidisciplinary rounds (*P* = .027), and their attitudes about it not being their role (*P* = .001)

**Table 1.** Summary of survey responses initial launch (2020) and postlaunch (2021 to 2023)

Questions asked		**Initial launch (** ***n*** **= 315) (%)**	**Postlaunch (** ***n*** **= 213) (%)**	**Difference between groups** ***P*** **-value**
Participant characteristics: RN years	<3 years	21	27	.191
	4–6 years	12	20	.030
	7–10 years	16	18	.615
	>10 years	51	35	.024
Participant view/attitude about participation in antibiotic stewardship	Should be involved	82	77	.644
	Not enough time but interested	15	13	.619
	Not my role	3	10	.001
Barriers to participation	Lack of knowledge	44	39	.450
	Culture of unit	11	15	.245
	No obstacles	36	34	.734
	Not my responsibility	9	12	.267
Patient rounding takes place on unit	Yes	65	79	.160
	No	35	21	.011
Comfortable participating in rounding	Yes	68	91	.027
Overall usefulness of online modules	Apply knowledge to current work	97	99	.877
	Empowers them to participate in rounding	73	78	.629

## Discussion

The pandemic presented unforeseen challenges for hospitals, including increased burden of care, fluctuations in staffing, supply shortages, and clinician turnover. Momentum was lost for process improvement projects. Antibiotic stewardship, especially in the early days of the pandemic, suffered from overuse of antibiotics in the emergency department, despite their lack of direct activity against the virus.^4^ Treatment options were limited early on, and guidance changed often as novel approaches to treatment were introduced.

Results of the survey taken by new hires throughout the pandemic showed a change in the level of the nurses’ experience which may be expected as many nurses left the workforce or retired. New hires came with their own experiences and attitudes and although the percentage was small that answered “not my role,” it was significant. The increase in number of units where nurses participated in multidisciplinary rounds increased by 14% but was not significant. This hospital had been rolling out the process of rounding unit by unit, well before the pandemic which may be in part responsible. Lastly, the nurse’s comfort level with participating in rounds was significant. This can be from increased knowledge as provided by the modules, general expectations of the culture itself, or previous experience with rounding.

Since the publication of our earlier work, there have been several studies that assessed nurses’ attitudes/behaviors and barriers toward participation in AS.^5–7^ They corroborated our earlier finding that education remains a barrier to participation in AS and that nurses felt they should be a part of the AS team. One recent pilot study used the premise that standardized education for nurses on principles of AS would improve their knowledge and confidence about AS.^7^ It suggested that this might motivate participation in AS activities.

Our most recent results continue to corroborate these findings. Healthcare systems should continue to explore ways to deliver AS awareness and education to nurses in formats that are convenient.^8^ Online learning provides such a method as it can be assigned or accessed as needed. Lessons learned around nursing education during the pandemic should be evaluated and studied for future planning.
